# The impact of affective and negative symptoms on the development of psychosis in a six-year follow-up of a community-based population

**DOI:** 10.1192/j.eurpsy.2024.515

**Published:** 2024-08-27

**Authors:** C. Ergul, T. Binbay, U. Kırlı, H. Elbi, K. Alptekin, J. van Os, M. Drukker

**Affiliations:** ^1^Maastricht University, Maastricht, Netherlands; ^2^Dokuz Eylul University; ^3^Ege University, Izmir, Türkiye; ^4^Utrecht University, Utrecht, Netherlands

## Abstract

**Introduction:**

The Clinical High Risk (CHR) group for transition to psychotic disorders (PD) is usually defined by the severity of positive symptoms, help-seeking and impairment in level of functioning. However, the CHR concept has a limited transition risk to PD. Recent studies have shown that some of the risks might be attributable to other symptoms.

**Objectives:**

This study investigates the association between affective and negative symptoms and the risk of transition to PD in a community-based population of 2185 participants in Turkey.

**Methods:**

At baseline, psychotic and affective symptomatology were assessed. The same participants were contacted again 6-years later. The initial analysis aimed to assess the link between affective and negative symptoms, and the progression to PD. The independent variable, baseline symptomatology, was categorized into five groups: no Psychotic Experiences (PE)(reference), subclinical PE, subclinical PE accompanied by affective/negative symptoms, clinical PE, and clinical PE with affective/negative symptoms. In the subsequent analysis, the association between affective and negative symptoms at baseline and the onset of PE and PD at follow-up was evaluated. For this analysis, the baseline symptomatology was restructured into two categories: neither PE nor affective/negative symptoms (reference), and the presence of affective/negative symptoms without PE.

**Results:**

The findings from the initial analysis indicated that being part of the ‘subclinical PE only’ group at baseline was not associated with an increased risk of developing PD at follow-up. Being part of the ‘subclinical PE+affective/negative symptoms’ group was not significantly associated with PD at follow-up, although a trend was observed (OR: 3.22; z=1.90; p=0.057). Moreover, being classified as having ‘clinical PE only’ (OR: 6.23; z=2.57; p=0.010) and ‘clinical PE+affective/negative symptoms’ (OR: 8.48; z=4.17; p=0.001) at baseline was associated with an increased risk of developing PD at follow-up. Results from the subsequent analysis showed that being in the ‘affective/negative symptoms’ group at baseline was associated with an increased risk of new subclinical PE (RR: 1.98; z=3.20; p=0.001), new clinical PE (RR: 3.14; z=4.84; p=0.001), and new PD (RR: 4.21; z=2.17; p=0.030) at follow-up, compared to the ‘neither PE nor affective/negative symptoms’ group.

**Conclusions:**

The results confirm that baseline severity of positive symptoms is significant in predicting transition to PD. In addition, the findings imply that not only positive symptoms but also affective and negative symptoms might contribute to the risk of transition to PD as well as incident psychotic symptoms. Defining CHR groups based on a combination of positive, affective and negative symptoms instead of focusing only on positive symptoms likely will help more accurately predict the transition to psychosis.

**Disclosure of Interest:**

None Declared

